# Long‐Term Bone Height Changes After Sinus Floor Elevation With Maxillary or Mandibular Bone Grafts: A Radiological Study

**DOI:** 10.1111/cid.70008

**Published:** 2025-02-06

**Authors:** Wilhelmus F. Bouwman, Francis A. Eijsackers, Nathalie Bravenboer, Christiaan M. ten Bruggenkate, Sharon Remmelzwaal, Engelbert A. J. M. Schulten

**Affiliations:** ^1^ Oral and Maxillofacial Surgeon, Department of Oral and Maxillofacial Surgery/Oral Pathology, Amsterdam UMC and Academic Centre for Dentistry Amsterdam (ACTA) Vrije Universiteit Amsterdam, Amsterdam Movement Sciences Amsterdam The Netherlands; ^2^ Research Analyst, Francis4YourPractice Leiden The Netherlands; ^3^ Department of Laboratory Science, Amsterdam UMC Vrije Universiteit Amsterdam, Amsterdam Movement Sciences Amsterdam The Netherlands; ^4^ Department of Epidemiology and Data Science Amsterdam UMC—VUmc Amsterdam The Netherlands

**Keywords:** atrophic maxilla, autogenous bone graft, long‐term study, mandibular bone grafts, maxillary bone grafts, maxillary sinus floor elevation, radiographs, radiological measurements, sinus augmentation, sinus floor elevation

## Abstract

**Background:**

This retrospective study aimed to assess the impact of maxillary and mandibular autogenous bone grafts on maxillary bone height in patients who underwent maxillary sinus floor elevation (MSFE).

**Methods:**

The study involved 92 patients, divided into two groups: 37 patients receiving maxillary bone grafts for MSFE and 55 patients receiving mandibular bone grafts for MSFE. Bone height after MSFE was measured on panoramic radiographs up to 60 months postoperatively, comparing different positions and situations.

**Results:**

In general, both maxillary and mandibular bone grafts resulted in an increase in bone height directly after the MSFE procedure, followed by a bone height loss over time, with no significant differences between the two groups for gap and free‐end positions. However, at distal to implant positions, mandibular bone grafts showed less bone loss. Despite gradual bone height loss, all implants remained covered with bone without exposure or subsequent loss, indicating a successful MSFE procedure.

**Conclusion:**

This radiologic study showed that over a long‐term period there is a similar bone height pattern at dental implant sites and sites distal to implants when maxillary or mandibular bone grafts are used in MSFE.

**Abbreviations**
ANOVA: Analysis of variance
BCP: biphasic calcium phosphate
Beta‐TCP: β‐tricalcium phosphate
BIC: bone to implant contact
CaP: calcium phosphate
g: gram
h: hour
mg: milligram
mL: milliliter
mm: millimeters
MSFE: maxillary sinus floor elevation
ns: not significant
*p*‐value: null hypothesis significance testing
SD: standard deviation
SLA: sandblasted, large‐grit, acid‐etched
w/w: weight‐based

## 1 Introduction

Maxillary sinus floor elevation (MSFE) is a frequently performed pre‐implant surgical procedure and is regarded as a safe technique for restoring insufficient alveolar bone height in the posterior maxilla to allow dental implant placement for dental rehabilitation. Extraction of one or more teeth frequently results in atrophy of the alveolar ridge and pneumatization of the maxillary sinus [1, 2]. Augmentation of the maxillary sinus floor can be performed with autogenous bone grafts, xenogenic bone substitutes (or a mixture of autogenous bone grafts and xenogenic bone substitutes), allograft bone, or alloplastic materials [3]. Currently, the ideal grafting material for MSFE has not yet been found; ideally, a bone substitute is totally replaced by bone [4, 5, 6, 7]. Therefore, and for surgical convenience, bone substitutes are frequently used as graft material [8, 9]. However, bone substitutes only provide an osteoconductive scaffold [10]. The replacement of a bone substitute graft to bone takes time [8, 11]. Even with longer healing times (9–12 months), not all bone substitute will be resorbed and replaced by vital bone [11]. Velich et al. investigated the behavior of several bone substitutes in MSFE: autogenous bone, a calcium carbonate‐coated polymer, hydroxyapatite (HA) of algal origin, calcium carbonate gel produced from coral or beta‐tricalcium phosphate alone, autogenous bone mixed with these bone substitutes, or a combination of beta‐tricalcium phosphate and platelet‐rich plasma. Total resorption (disappearance) of the bone substitute material was observed in only 2.7% of the cases. This lack of resorption was similar for most bone substitutes [12]. Autogenous bone is frequently used in MSFE due to its superior osteogenic, osteoinductive, and osteoconductive properties. It is often considered the gold standard for bone grafting because it contains live cells and growth factors that enhance bone regeneration. Specific indications for using autogenous bone in MSFE include: (1) severely atrophic maxillary sinus (< 4 mm), autogenous bone may be required to ensure proper regeneration and stability for dental implant placement; (2) need for rapid bone regeneration due to faster healing and integration compared to some synthetic or allogenic grafts; (3) compromised bone quality (type IV bone); autogenous bone can improve the regenerative environment; (4) large defects or extensive augmentation; (5) previous graft failure; autogenous bone may be used to enhance outcomes; (6) high risk of infection or poor healing, growth factors, and living cells, which can enhance the healing response and reduce the risk of infection, such as diabetes mellitus; (7) requirement for a graft with osteogenic properties, as autogenous bone contains live osteoblasts and osteoprogenitor cells [3].

The utilization of autogenous bone grafts requires an additional surgical intervention, which carries the potential for complications and adverse effects at the donor site [13, 14, 15, 16, 17]. Autogenous bone grafts can be harvested from various extraoral or intraoral donor sites, for instance the iliac crest, the symphyseal or retromolar regions of the mandible, or the maxillary tuberosity. Grafting from different intraoral sites has the advantages of local rather than general anesthesia, a short distance between the donor and augmentation sites, and the avoidance of cutaneous scars. It has been demonstrated that bone grafts collected from intraoral sites other than the chin result in a much higher total bone volume (TBV) than iliac crest bone [18]. There is no comparative evidence on the impact of different donor sites for autogenous bone grafts on bone regeneration, despite the possibility of changes in bone loss and bone induction [19]. Loss of bone height after an initial increase in height is a common phenomenon that occurs with both autogenous bone grafts and alloplastic materials [20]. Both the fraction of HA within the calcium phosphate ceramics and loading of the implant appear to be advantageous for tissue height maintenance after MSFE [21]. According to several authors, it is inevitable for grafted material to experience a decrease in height due to the air pressure caused by respiration in the maxillary sinus [22, 23]. Radiographic results indicate that bone substitutes used in MSFE procedures are superior to autogenous bone grafts in similar types of surgery [20].

Non‐loaded bone seems to be particularly susceptible to resorption [24]. As resorption‐preventing elements, dental implants are crucial as loaded pillars within the bone structure. As such, dental implants provide support for three‐dimensional (vertically, longitudinally, and labial/lingual) reconstruction [25]. Several publications mention bone scalloping around the “apices” of dental implants, implying that bone resorbs more rapidly in non‐loaded areas than in loaded parts or as a result of pneumatization of the maxillary sinus [26]. There have been no studies, as far as the authors know, on the use of exclusively autogenous bone grafts from the maxilla or the mandible in MSFE, with follow‐up lasting up to 5 years.

The aim of this study was to investigate the radiological changes in bone height after MSFE using two types of autogenous bone grafts with a follow‐up time of 60 months after dental implant placement. In particular, the authors focused on the bone height reduction in implant‐ and distal to implant positions as well as gap‐ and free‐end situations after MSFE.

## 2 Materials and Methods

### 2.1 Patients

This retrospective study is based on data collected from medical records (chart reviews) and panoramic radiographs that were obtained during patients' annual follow‐up visits. Between 9/1999 and 12/2013 a total of 313 consecutive patients (146 males and 167 females) with a mean age of 55 years (range: 29–81) who underwent an MSFE using intraoral bone grafts in a (partially) posterior edentulous maxilla were included. When a substantial quantity of autogenous bone graft was necessary for space expansion in MSFE, it was imperative to extract this larger amount of bone from the mandible rather than the maxilla. Based on patients' specific anatomical conditions, 96 of the 313 patients had received maxillary bone grafts, and 217 patients had received mandibular bone grafts. The 313 patients had received a total of 482 dental implants.

A native alveolar bone height of less than 8 mm in the posterior edentulous maxilla along with the patient's preference for oral rehabilitation with dental implants served as criteria for treatment selection. Patients with a history of radiation of the jaws, heart valve prostheses, or endocarditis were excluded from dental implant surgery. Smokers (14.1%) were not excluded from dental implant surgery. Prior to both surgical procedures (MSFE and dental implant surgery), all patients signed a written consent for the use of their data and received standard care. The MSFE procedure, dental implant placement, and the panoramic radiograph measurements were conducted at the Department of Oral and Maxillofacial Surgery/Oral Pathology, Amsterdam UMC (location VUmc), Amsterdam, The Netherlands. For the purpose of this study, the following patients were excluded: patients with analog radiographs, poor‐quality radiographs, patients with simultaneous buccal bone augmentation, patients with MSFE and simultaneous dental implant placement, fully edentulous patients, and patients with extra‐orally harvested bone grafts.

Due to the incomplete participation of the 313 patients in routine annual examinations, only those with adequate follow‐up (at least a minimum of five timepoints after dental implant placement) for radiological measurements were included, yielding a total of 92 patients at the start of the study. Among these, 54 patients were observed for up to 48 months and 40 patients were observed for up to 60 months. A total of 95 treatments were administered to the 92 patients, as three individuals underwent surgery on both sites during two separate treatment sessions. All patients received autogenous graft material.

The study was conducted according to the guidelines of the Declaration of Helsinki and approved by the Medical Ethics Review Committee of VU University Medical Center (registration number: 2021.5723). The study is registered with the US Office for Human Research Protections (OHRP) as IRB00002991. The FWA number assigned to VU University Medical Center is FWA00017598.

### 2.2 Maxillary Sinus Floor Elevation and Bone Harvesting Procedures

All 92 patients underwent an MSFE procedure, according to Tatum's top‐hinge trapdoor technique [2]. During the perioperative period, prophylactic procedures were carried out as previously published [21]. The maxillary tuberosity bone grafts were harvested with a mallet and an osteotome. The bone grafts from the mandible and chin (one patient) were harvested in a half‐cylinder shape with explantation trephines (inner diameter 4.2 mm; Institute Straumann AG, Basel, Switzerland), with a drilling speed of 500 rpm, using sterile saline for copious irrigation. The bone grafts from the maxilla, mandible, or chin were positioned underneath the lifted trapdoor and Schneiderian membrane without exerting significant pressure on the lifted anatomical structures. No barrier membranes were used to cover the lateral window [27]. The wounds were closed with Gore‐Tex sutures (W.L. Gore & Associates, Newark, DE, USA). The sutures were removed after 10–14 days.

### 2.3 Dental Implant Placement

In all included 92 patients, 158 Straumann SLA Soft Tissue Level dental implants with diameters of 3.3 mm or 4.1 mm and implant lengths of 10 or 12 mm were placed (Institut Straumann AG, Basel, Switzerland). According to the manufacturer's instructions, dental implant surgery was performed 4 months after MSFE. During the perioperative period, prophylactic procedures were carried out as previously published [21]. During the 3‐month osseointegration period, the implants were not loaded, and the restorative dentist subsequently performed the prosthetic treatment.

### 2.4 Radiological Evaluation

**FIGURE 1:**
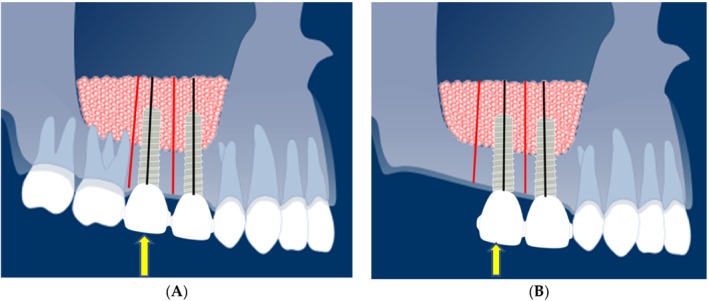
(A) This graphic shows the locations of the bone height measurements at the implant and distal to implant positions. The black lines represent the measurement positions at implant sites; the red lines (2–3 mm distally) represent the “distal” or intermediate positions. The yellow arrows point to the most distal implant positions. (B) This graphic shows the locations of the bone height measurements at the implant and distal to implant positions (free‐end situation). The black lines represent the measurement positions at implant sites, while the red lines (2–3 mm distally) represent the “distal” positions (free‐end situation). *Source:* Graphics used with courtesy of the ITI Foundation, Basel, Switzerland.

Considering regional differences in mechanical loading of the grafted bone and the effect of loading on alveolar bone height gain, bone height was measured at the distal positions of distal dental implants in gap situations (Figure 1A) and in free‐end situations (Figure 1B).

Panoramic radiographs were taken at the following timepoints: (T0) patient intake, approximately 2 months prior to MSFE; (T1) immediately after MSFE; (T2) approximately 1 month prior to the dental implant placement; (T3) immediately after dental implant placement; (T4) after an osseointegration period of 3 months; and (T5‐T9) during annual follow‐up recall visits up to 60 months after dental implant placement.

Bone heights were measured on the panoramic radiographs at the dental implant sites and 2–3 mm distally of the dental implant sites (as an inter‐implant site, from now on referred to as gap situations, or as a free‐end distal measurement site) and corrected for the magnification factor of 1.25, according to the method described by Zijderveld et al. [20] (Figure 1A,B).

To prevent miscalculation of the cranial bone levels, a correction was made for the possibility of peri‐implant bone loss in the cervical region.

### 2.5 Statistical Analysis

The data are expressed as the mean and standard deviation between brackets. Because the bone heights of the same individual were measured multiple times (T0–T9) and 158 implants were measured in 92 patients, a one‐way repeated measures (ANOVA) was used to account for the non‐independence in the data. Maximum bone height loss was calculated at T8 (T1–T8) as well as T9 (T1–T9), and the difference between the two timepoints was examined using a paired *t*‐test. In addition, differences between implant sites and distal to implant positions were also determined by a paired *t*‐test. Differences between maxillary and mandibular bone, as well as gap‐ and free‐end situations, were evaluated using an independent sample *t*‐test; *p* < 0.05 was considered significant. Since patients were treated exclusively with either maxillary or mandibular bone, within‐patient testing is not feasible. Version 28 of IBM SPSS was used for all statistical analyses.

This study is in compliance with the STROBE guidelines.

## 3 Results

### 3.1 Patient Population

The patient data were divided into two groups: the first group with maxillary bone grafts consisted of 37 patients (17 males and 20 females with a mean age of 57 years) who received 64 dental implants, and the second group with mandibular bone grafts consisted of 55 patients (23 males and 32 females with a mean age of 54 years) who received 94 dental implants (Tables 1 and 2). At T8 and T9, 54 (60% of the total population), and 40 patients (44% of the total population), respectively, returned for regular follow‐up.

**TABLE 1:** Patient data for maxillary sinus floor elevation (MSFE) is divided into two groups: Maxillary bone grafts and mandibular bone grafts. M—male; F—Female.

Grafts harvested from	Maxilla	Mandible
Number of patients	37	55
Number of dental implant sites	64	94
Gender	17 M, 20 F	23 M, 32 F
Mean age and range in years	57 (29–81)	54 (30–74)
Mean observation period in months	33	43

**TABLE 2:** Number of available measurements at multiple time intervals up to 60 months of follow‐up.

Time points	Months of observation	Maxillary bone grafts	Mandibular bone grafts	Total implants observed	Number of patients
T0	–6	64	94	158	92
T1	–4	58	94	152	88
T2	−1	54	83	137	81
T3	0	64	94	158	92
T4	3	44	89	133	82
T5	12	56	79	135	80
T6	24	42	78	120	70
T7	36	35	67	102	61
T8	48	24	70	94	54
T9	60	28	41	69	40

*Note:* T0: Patient intake; T1: Maxillary sinus floor elevation; T2: Ridge mapping; T3: Dental implant placement; T4: Prosthetic loading; T5: Follow‐up visit after 12 months; T6: Follow‐up visit after 24 months; T7: Follow‐up visit after 36 months; T8: Follow‐up visit after 48 months; T9: Follow‐up visit after 60 months. A part of the total patient population neglected to appear at various timepoints for a panoramic radiograph.

### 3.2 Maxillary and Mandibular Bone Grafts

**FIGURE 2:**
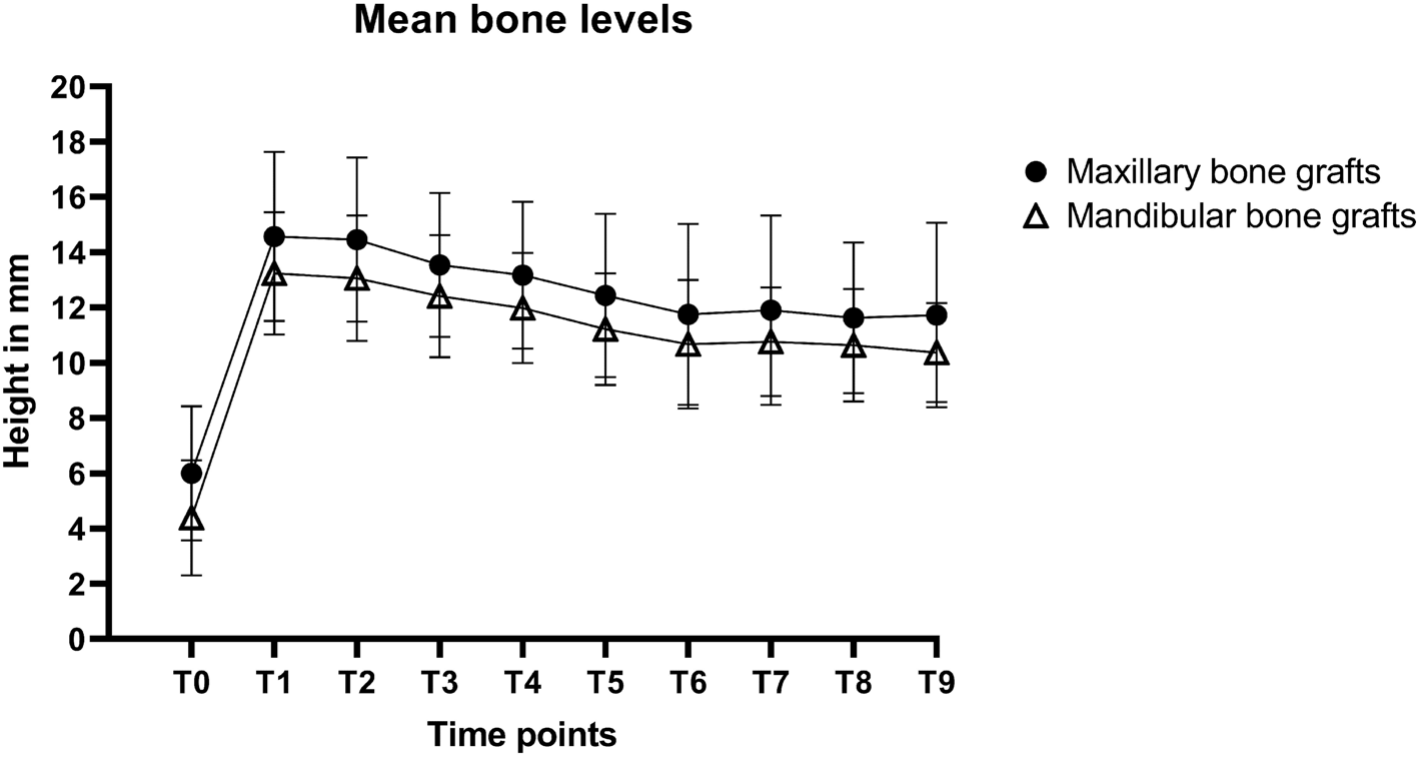
Mean bone height measurements (and standard deviations) of maxillary sinus floor elevation (MSFE) with maxillary or mandibular bone grafts and 60‐month follow‐up after dental implant placement T0: Patient intake; T1: Maxillary sinus floor elevation; T2: Ridge mapping; T3: Dental implant placement; T4: Prosthetic loading; T5: Follow‐up visit after 12 months; T6: Follow‐up visit after 24 months; T7: Follow‐up visit after 36 months; T8: Follow‐up visit after 48 months; T9: Follow‐up visit after 60 months.

As shown in Figure 2 and Table 3, the mean alveolar bone height at T0 was larger in the maxillary bone graft group (6.0 mm) than in the mandibular bone graft group (4.4 mm) (*p* < 0.001). The initial bone height gain after MSFE at T1 was comparable (8.7 mm in the maxillary bone graft group versus 8.9 mm in the mandibular bone graft group, *p* = 0.643). The total bone height loss from T1 to T8 was similar in the maxillary and mandibular bone graft groups, with losses of 2.8 and 2.6 mm (*p* = 0.652), respectively. The total bone height loss at T9 was also similar in both bone graft groups: 2.9 mm in the maxillary and 2.3 mm in the mandibular grafts (*p* = 0.155).

**TABLE 3:** Radiographic bone height measurements (and standard deviations) of maxillary sinus floor elevation (MSFE) with maxillary bone grafts and mandibular bone.

		Maxillary bone grafts in mm (mean ± SD)	Mandibular bone grafts in mm (mean ± SD)
Time points	Months of observation	Mean bone height levels	Mean bone height levels
T0	−6	6.0 (2.4)	4.4 (2.1)
T1	−4	14.6 (3.1)	13.2 (2.2)
T2	−1	14.5 (3.0)	13.1 (2.3)
T3	0	13.5 (2.6)	12.4 (2.2)
T4	3	13.2 (2.7)	12.0 (2.0)
T5	12	12.4 (3.0)	11.2 (2.0)
T6	24	11.8 (3.3)	10.7 (2.3)
T7	36	11.9 (3.5)	10.8 (2.0)
T8	48	11.6 (2.8)	10.6 (2.0)
T9	60	11.7 (3.4)	10.4 (1.8)

*Note:* T0: Patient intake; T1: Maxillary sinus floor elevation; T2: Ridge mapping; T3: Dental implant placement; T4: Prosthetic loading; T5: Follow‐up visit after 12 months; T6: Follow‐up visit after 24 months; T7: Follow‐up visit after 36 months; T8: Follow‐up visit after 48 months; T9: Follow‐up visit after 60 months.

### 3.3 Dental Implant Sites and Distal to Implant Positions

**FIGURE 3:**
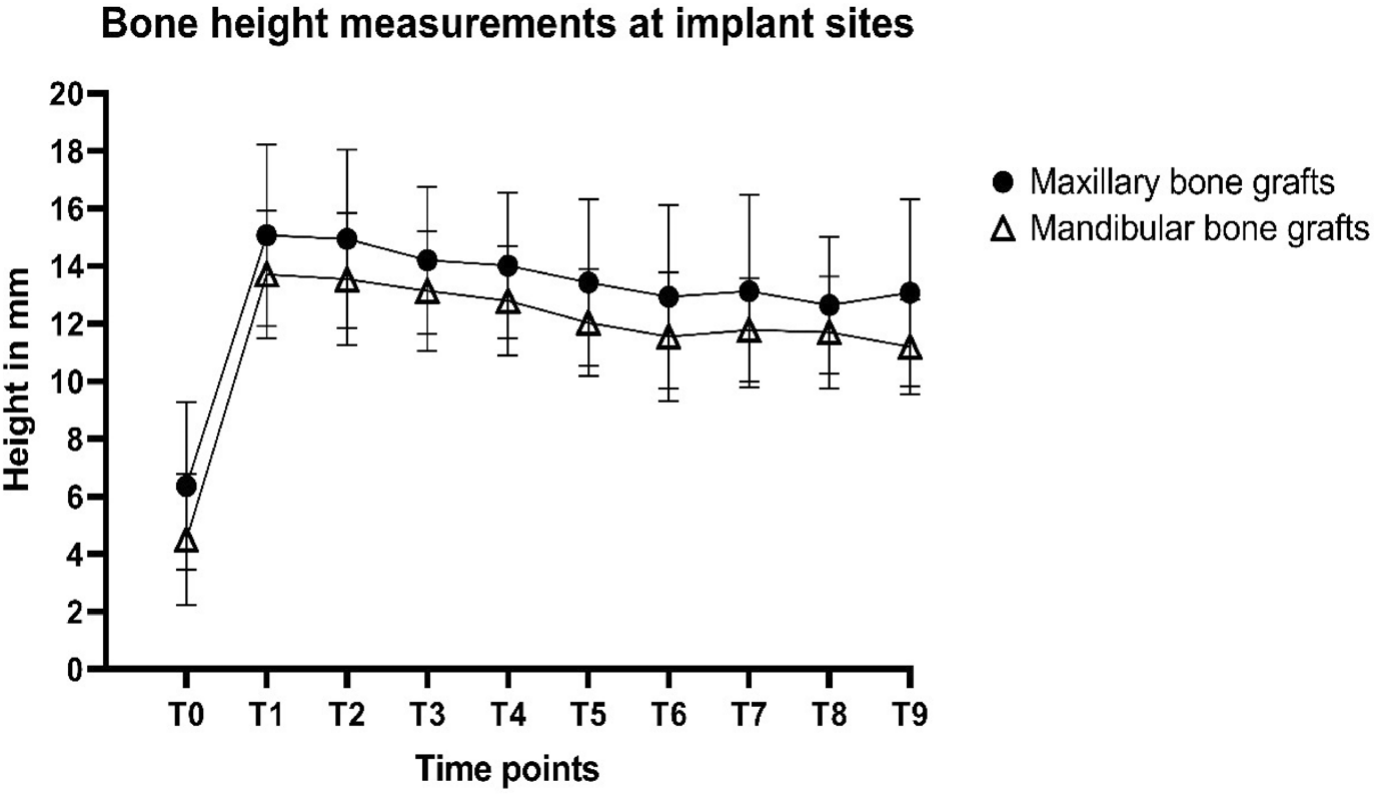
Mean bone height measurements at implant sites (and standard deviations) of maxillary sinus floor elevation (MSFE) with maxillary or mandibular bone grafts and 60‐month follow‐up after dental implant placement T0: Patient intake; T1: Maxillary sinus floor elevation; T2: Ridge mapping; T3: Dental implant placement; T4: Prosthetic loading; T5: Follow‐up visit after 12 months; T6: Follow‐up visit after 24 months; T7: Follow‐up visit after 36 months; T8: Follow‐up visit after 48 months; T9: Follow‐up visit after 60 months.

**FIGURE 4:**
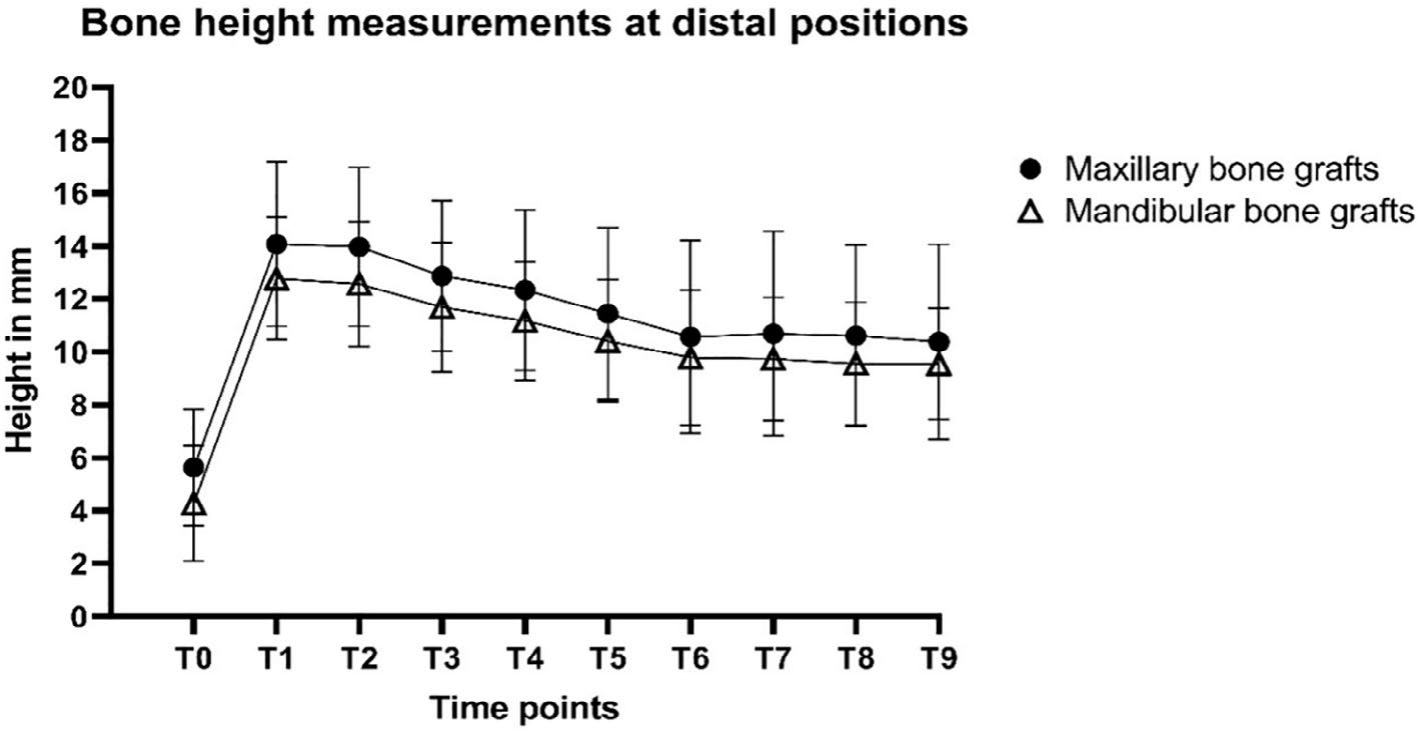
Mean bone height measurements at distal to implant positions (and standard deviations) of maxillary sinus floor elevation (MSFE) with oral bone grafts with a 60‐month follow‐up after dental implant placement T0: Patient intake; T1: Maxillary sinus floor elevation; T2: Ridge mapping; T3: Dental implant placement; T4: Prosthetic loading; T5: Follow‐up visit after 12 months; T6: Follow‐up visit after 24 months; T7: Follow‐up visit after 36 months; T8: Follow‐up visit after 48 months; T9: Follow‐up visit after 60 months.

The total bone height loss at implant sites calculated at T8 (T1–T8) did not differ significantly between the two groups (*p* = 0.487), with a mean of 2.5 mm for the maxillary bone graft group and 2.0 mm for the mandibular bone graft group (Figure 3 and Table 4). At T9 (T1–T9), the total bone height loss at implant sites (both maxillary and mandibular) was not statistically significant, with a mean of 2.0 mm in the maxillary group and 2.5 mm in the mandibular group (*p* = 0.968). The total bone height loss at the distal to implant position at T8 was not statistically different between the two groups (*p* = 0.882), with a mean of 3.5 mm in the maxillary bone graft group and 3.2 mm in the mandibular bone graft group (Figure 4 and Table 4). Statistically, the total bone height loss at distal to implant positions was significantly different between the two groups at T9 (*p* = 0.023), with a mean of 3.7 mm in the maxillary bone graft group and 3.3 mm in the mandibular bone graft group.

**TABLE 4:** The radiographic bone level measurements (and standard deviations) of 158 maxillary sinus floor elevations (MSFE).

Time points	Months of observation	Autogenous bone grafts implant sites in mm (mean ± SD)		Autogenous bone grafts distal to implant positions in mm (mean ± SD)	
		Maxillary	Mandibular	Maxillary	Mandibular
T0	−6	6.4 (2.9)	4.5 (2.3)	5.6 (2.2)	4.3 (2.2)
T1	−4	15.1 (3.2)	13.7 (2.2)	14.1 (3.1)	12.8 (2.3)
T2	−1	14.9 (3.1)	13.5 (2.3)	14.0 (3.0)	12.6 (2.3)
T3	0	14.2 (2.6)	13.1 (2.1)	12.9 (2.8)	11.7 (2.5)
T4	3	14.0 (2.5)	12.8 (1.9)	12.3 (3.0)	11.2 (2.2)
T5	12	13.4 (2.9)	12.0 (1.9)	11.4 (3.2)	10.4 (2.3)
T6	24	12.9 (3.2)	11.6 (2.3)	10.6 (3.7)	9.8 (2.6)
T7	36	13.1 (3.4)	11.8 (1.8)	10.7 (3.9)	9.7 (2.3)
T8	48	12.6 (2.4)	11.7 (1.9)	10.6 (3.4)	9.6 (2.3)
T9	60	13.1 (3.3)	11.2 (1.7)	10.4 (3.7)	9.5 (2.1)

*Note:* T0: Patient intake; T1: Maxillary sinus floor elevation; T2: Ridge mapping; T3: Dental implant placement; T4: Prosthetic loading; T5: Follow‐up visit after 12 months; T6: Follow‐up visit after 24 months; T7: Follow‐up visit after 36 months; T8: Follow‐up visit after 48 months; T9: Follow‐up visit after 60 months; initial height gain is T1–T0; final height gain is T9–T0.

### 3.4 Gap and Free‐End Situation

Table 5 shows the total number of dental implants placed in gap situations (24 patients) and free‐end situations (71 patients) after MSFE with maxillary or mandibular bone grafts.

**TABLE 5:** Absolute number of maxillary sinus floor elevations with maxillary and mandibular bone grafts according to the number of gap and free‐end situations and dental implants.

	Gap situation/dental implants	Free‐end situation/dental implants	Total of MSFE/dental implants
Maxillary bone graft	7/11	33/53	40/64
Mandibular bone graft	17/30	38/64	55/94
Total	24/41	71/117	95(*)/158

*Note:* *95 treatments were administered to 92 patients.

**FIGURE 5:**
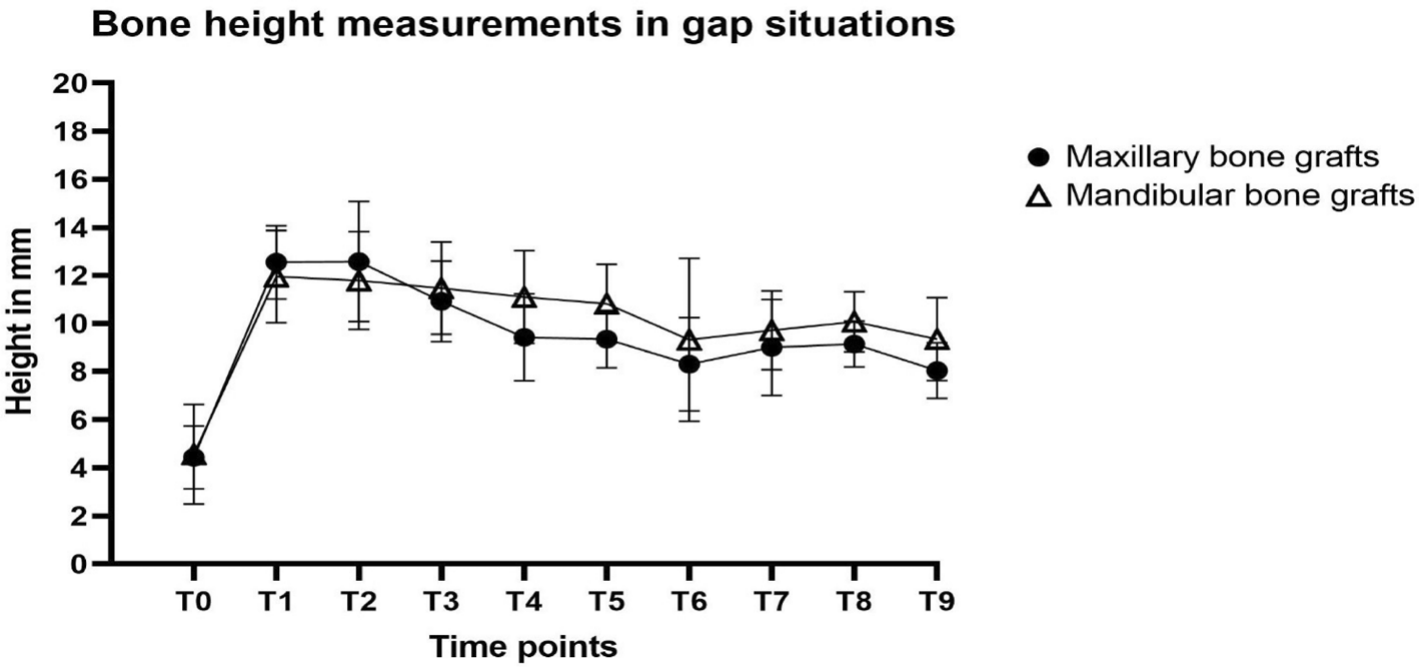
Mean bone height measurements in gap situations (and standard deviations) of maxillary sinus floor elevation (MSFE) with maxillary or mandibular bone grafts and 60‐month follow‐up after dental implant placement T0: Patient intake; T1: Maxillary sinus floor elevation; T2: Ridge mapping; T3: Dental implant placement; T4: Prosthetic loading; T5: Follow‐up visit after 12 months; T6: Follow‐up visit after 24 months; T7: Follow‐up visit after 36 months; T8: Follow‐up visit after 48 months; T9: Follow‐up visit after 60 months.

**FIGURE 6:**
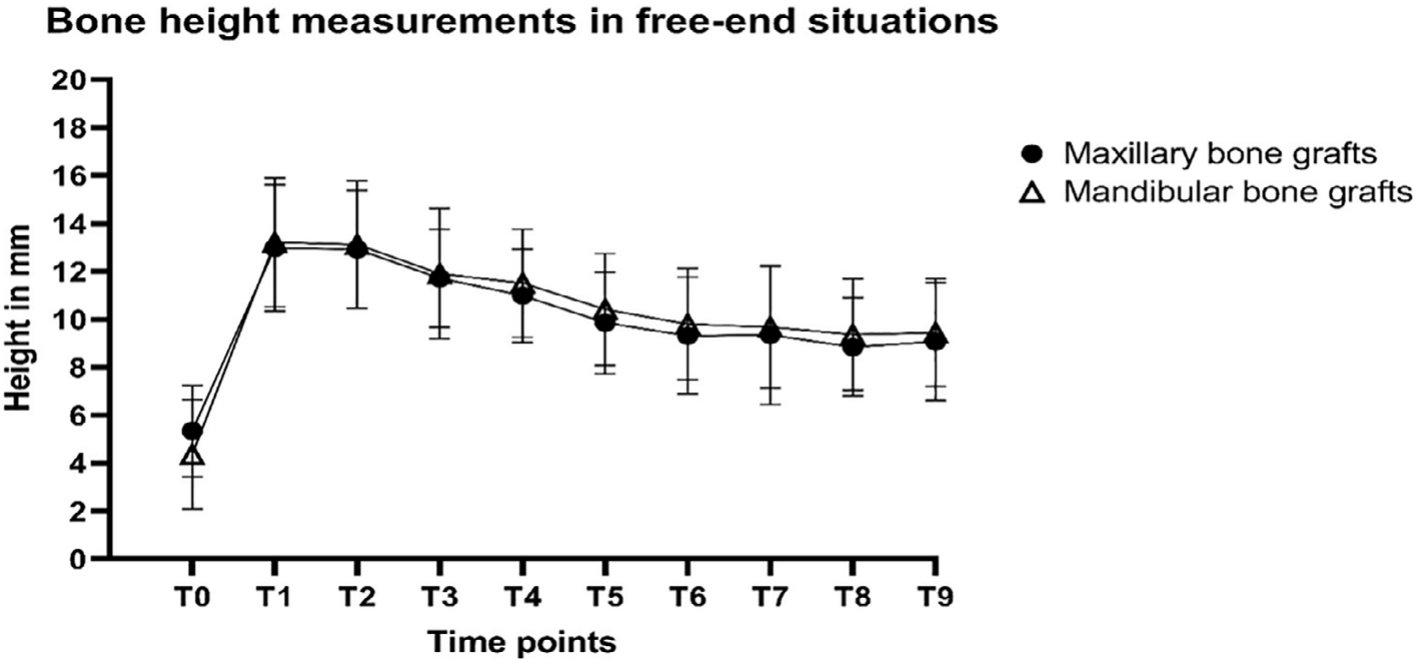
Mean bone height level measurements in free‐end situations (and standard deviations) at distal to implant sites. T0: Patient intake; T1: Maxillary sinus floor elevation; T2: Ridge mapping; T3: Dental implant placement; T4: Prosthetic loading; T5: Follow‐up visit after 12 months; T6: Follow‐up visit after 24 months; T7: Follow‐up visit after 36 months; T8: Follow‐up visit after 48 months; T9: Follow‐up visit after 60 months.

Total bone height loss was not different for maxillary versus mandibular bone grafts in gap situations for both T8 and T9 (T8: 2.3 vs. 2.0 mm, *p* = 0.768; T9: 3.7 vs. 1.9 mm, *p* = 0.07) (Figure 5 and Table 6). Similar patterns were observed for free‐end situations with losses at T8 of 3.5 and 3.8 mm (*p* = 0.808) and at T9 of 3.9 and 3.0 mm (*p* = 0.335) in maxillary and mandibular bone grafts, respectively (Figure 6 and Table 6).

**TABLE 6:** Bone level measurements (and standard deviations) at distal to implant sites in gap situations and in free‐end situations.

Time points	Months of observation	Distal mean bone levels in gap situations in mm (mean SD)		Distal mean bone levels in free‐end situation in mm (mean SD)	
		Maxillary bone grafts	Mandibular bone grafts	Maxillary bone grafts	Mandibular bone grafts
T0	−6	4.4 (1.3)	4.6 (2.1)	5.4 (1.9)	4.4 (2.3)
T1	−4	12.6 (1.5)	12.0 (1.9)	13.0 (2.6)	13.2 (2.7)
T2	−1	12.6 (2.5)	11.8 (2.0)	12.9 (2.5)	13.1 (2.7)
T3	0	10.9 (1.7)	11.5 (1.9)	11.7 (2.0)	11.9 (2.7)
T4	3	9.4 (1.8)	11.1 (1.9)	11.0 (2.0)	11.5 (2.3)
T5	12	9.3 (1.2)	10.8 (1.6)	9.9 (2.1)	10.4 (2.3)
T6	24	8.3 (1.9)	9.3 (3.4)	9.3 (2.4)	9.8 (2.3)
T7	36	9.0 (2.0)	9.7 (1.6)	9.4 (2.9)	9.7 (2.5)
T8	48	9.2 (1.0)	10.1 (1.3)	8.8 (2.1)	9.4 (2.3)
T9	60	8.0 (1.2)	9.4 (1.7)	9.1 (2.5)	9.4 (2.2)

*Note:* T0: Patient intake; T1: Maxillary sinus floor elevation; T2: Ridge mapping; T3: Dental implant placement; T4: Prosthetic loading; T5: Follow‐up visit after 12 months; T6: Follow‐up visit after 24 months; T7: Follow‐up visit after 36 months; T8: Follow‐up visit after 48 months; T9: Follow‐up visit after 60 months.

## 4 Discussion

This study evaluated the changes in bone height after MSFE using two types of autogenous bone grafts, one taken from the retromolar area of the mandible and the other from the maxillary tuberosity, over a prolonged period of time. This study showed that 48 and 60 months after MSFE, the total bone height, measured on panoramic radiographs, did not differ between maxillary or mandibular bone grafts at dental implant sites as well as at distal to implant sites. For distal to implant sites, bone height was comparable in both free‐end and gap situations. The total bone height was lower at distal positions compared to implant positions.

Our findings demonstrate a general trend of gradual bone height loss following the initial increase after the MSFE procedure, up to 60 months for both intraoral donor sites, which is consistent with our hypothesis. A similar pattern was observed in a study that used calcium phosphate ceramics as graft material [21]. In that study, three types of calcium phosphate ceramics were compared after MSFE (β‐TCP, BCP (20–80) 20% HA‐80% β‐TCP, and BCP (60–40) 60% HA‐40% β‐TCP), which revealed that after initial settling of the bone substitute particles in the maxillary sinus was followed by resorption of the grafted material for all three types of calcium phosphate ceramics, of which the majority of resorption occurred within the first 24 months and then appeared to slow down [21]. A gradual height loss after the initial increase was also observed by radiographic assessment with two other types of bone substitutes (Bio‐Oss and Cerabone), but they showed the greatest vertical resorption after 12 months (55%–65% of tissue height loss) and an annual height loss of approximately 10%–12% over a total of 48 months [28]. Height loss of grafted material due to the pressure from respiratory airflow in the maxillary sinus is unavoidable, according to some authors [22, 23]. Whether this is an additional explanation for the height loss (resorption) of the autogenous bone grafts used in the present study remains unclear. Nevertheless, as previously stated, autogenous bone is still the preferred material for MSFE and the “gold standard.” HA bone substitutes are widely used due to their limited solubility and because they are widely and easily commercially accessible. This characteristic is believed to prevent excessive graft resorption, thereby reducing the risk of implant apices becoming exposed.

In general, the MSFE procedure is performed to increase the height of the lateral maxilla, eventually facilitating the placement of dental implants. In our study, in all cases, sufficient bone height was achieved to allow the planned placement of dental implants with lengths of 10 or 12 mm. The reduction in bone height, after the initial increase, never reached a point in the course of 5 years where the implants were exposed or subsequently lost, which demonstrates that the intended purpose of the augmentation procedure was fulfilled.

Overall, our findings did not support a preferential origin of the autogenous bone grafts since differences could not be detected. In contrast, Wu et al. argue that based on histological investigation of the bone biopsies harvested at dental implant surgery 4 months after MSFE, maxillary bone grafts may perform better than mandibular bone grafts in MSFE due to enhanced vascularization, osteoid deposition, and active bone remodeling. The grafts were compared using histology and histomorphometry, which revealed the better vascularity of the maxillary bone grafts. However, this does not prove that the material is superior for dental implant placement in the long‐term. Due to the histological nature of the Wu et al. study, the influence of clinical aspects like bone density was not taken into account. The density of the graft material, which was omitted from this study, could have a substantial effect on the primary stability of dental implants placed after MSFE [29].

Our findings indicate that total bone height loss is greater at distal to implant positions compared to implant positions, regardless of the type of bone graft used. The higher bone loss probably occurs as a result of the lack of dental implant load on the graft material. These results emphasize the importance of ongoing monitoring and evaluation in implant dentistry to make informed decisions regarding graft material and implant location, ultimately optimizing the long‐term success and stability of dental implants.

The bone height in the maxillary and mandibular groups was significantly different at baseline. This difference could have influenced the final bone height. When MSFE requires a considerable quantity of autogenous bone graft for space expansion, the choice of graft is usually mandibular bone because the mandible provides a greater quantity of accessible bone compared to the maxilla. This difference is therefore unavoidable, but since both maxillary and mandibular bone grafts were used for native bone heights of at least 4 mm, taking into account the patient's anatomical conditions, the effect on the final bone height is probably rather small. The decisive factor was the targeted volume increase, which depended on whether there was sufficient bone available from the maxilla or whether bone from the mandible was required.

In the present study, bone heights were measured on panoramic radiographs. The relevance of radiographic measurements of MSFE with bone substitutes for the stability and longevity of dental implants is limited since they do not reveal the amount of vital implant‐supporting bone. Radiographic analysis does have the advantage of repeated measurements over time within one individual. This gives the opportunity to obtain data on bone height loss for a prolonged follow‐up.

A limitation of our study is the low number of measurements during the 60‐month follow‐up due to patient withdrawal. For this reason, an extra data collection point was chosen because fewer than 50% of the patients participated in the annual examination after 60 months. After 48 months, 60% of the patients remained in the population. Studies with a 48‐ to 60‐month follow‐up period are rare, and this study has yielded important insights regarding the use of autogenous bone grafts in MSFE procedures and dental implant placement. Another limitation of this study was that panoramic radiography was used. Cone beam computed tomography (CBCT) is currently more routinely used in research and clinical practice. CBCT provides three‐dimensional imaging of structures and other advantages over the traditional two‐dimensional panoramic view imaging, particularly for complex diagnostic and surgical applications. In particular, the resorption pattern of maxillary bone grafts in gap situations was discernible after prosthetic loading. Only nine patients who underwent MSFE with maxillary bone grafts were observed for the entire 60‐month period. Bone harvesting from the mandible for MSFE was performed more frequently based on the patient's anatomical condition. Despite the choice of bone grafts, there was no correlation between patient non‐attendance at the various time points and loss of bone height due to resorption.

The origin of the bone graft (maxilla or mandible) did not significantly affect the preservation of bone height around the implant site over a long‐term follow‐up. However, solely assessing bone height may not suffice to assess the longevity of dental implants after MSFE procedures.

## 5 Conclusion

This radiologic study showed that over a long‐term period there is a similar bone height pattern at dental implant sites and at distal to implant sites when maxillary or mandibular bone grafts are used in MSFE.

## Author Contributions

**Wilhelmus F. Bouwman:** conceptualization, data analysis, investigation, writing – original draft, writing – review and editing. **Francis A. Eijsackers:** data acquisition, visualization, writing – review and editing. **Nathalie Bravenboer:** conceptualization, methodology, writing – review and editing. **Christiaan M. ten Bruggenkate:** conceptualization, investigation, methodology, writing – review and editing. **Sharon Remmelzwaal:** data acquisition, data analysis, methodology, writing – review and editing. **Engelbert A.J.M. Schulten:** conceptualization, data analysis, supervision, writing – review and editing. All authors have read and agreed to the final version of the manuscript.

## Ethics Statement

The study was conducted according to the guidelines of the Declaration of Helsinki and approved by the Medical Ethics Review Committee of VU University Medical Center (registration number: 2021.5723). The study is registered with the US Office for Human Research Protections (OHRP) as IRB00002991. The FWA number assigned to VU University Medical Center is FWA00017598.

## Consent

Informed consent was obtained from all subjects involved in the study.

## Conflicts of Interest

The authors declare no conflicts of interest.

## Data Availability

The data that support the findings of this study are available on request from the corresponding author. The data are not publicly available due to privacy or ethical restrictions.
